# Editor’s Book Table

**Published:** 1870-01

**Authors:** 


### Editor's Book Table.

*A Practical Treatise on the Diseases of Children.* By ALFRED VOGEL, M.D., Professor of Clinical Medicine in the University of Dorpat, Russia. Translated and Edited by H. RAPHAEL, M.D., late House-Surgeon to Bellevue Hospital, Attending Physician to the Eastern Dispensary for the Diseases of Children, etc., etc. From the fourth German edition. Illustrated by Lithographic Plates. New York: D. Appleton & Company, 90, 92, and 94 Grand Street. 1870. Pp. 603. $4.50.

THIS book passed to its fourth German edition in eight years, and has been translated into all the prominent languages of Europe. The present translation is by permission, and the author kindly forwarded advance sheets of all new matter, or changes, in the last German edition.

In point of literary merit, the translation is a success; the inverted and otherwise idiomatic peculiarities of the original disappearing in a sufficiently transparent English. The name of the eminent publishing house upon the title page, is guarantee, in full, of the fact of its presentation to the American reader in a beautiful and suitable dress.

As a systematic treatise, it is unusually full and complete, as a glance at the table of contents and the very minute and extensive index, will demonstrate.

The first chapter is devoted to Anatamo-Pathological observations upon the infantile organism, discussed under the several heads: A, Respiration and Circulation; B, Secretions; C, Growth. Chapter II., gives General Rules for the Examination of Children — a most excellent *resumé*. Chapter III. treats of the Nursing and Care of Children, and is full of valuable hints and instructions. The remaining chapters are devoted to the consideration of special diseases.

We take unmingled pleasure in noticing that therapeutics, based on bald empiricism, find little place in our author's teachings.

A full account of the pathology, a clear, concise and accurate detail of symptoms, with all the known methods of differential diagnosis, prepare the way for suggestions in treatment of, generally, a satisfactory rational character. Polypharmacy finds little honor herein.

A book worthy our science and the age.

*On the Wasting Diseases of Infants and Children.* By EUSTACE SMITH, M.D., London, M.R.C.P., Physician Extraordinary to His Majesty the King of the Belgians; Physician to the N. W. London Free Dispensary for Sick Children, and to the Metropolitan Dispensary. Philadelphia: Henry C. Lea. 1870. Pp. 195.

WASTING, which is a sign of defective nutrition, is not always the first sign of a disease which may be detected, and may, even in some of the worst cases of defective nutrition, be entirely concealed from the superficial observer, by fatty degeneration or deposit, œdema, etc. Acute disease may be the starting point, or a great variety of hereditary, constitutional or acquired causes may be at the foundation, which any incidental cause may waken into full activity. This fact is always one of the chief sources of anxiety, pending the presence of intercurrent disorders, not, of themselves, seemingly threatening. *Obsta principiis* — sequela of diseases, not fatal, and from which recovery seems *almost* complete, should be thoroughly studied, lest, peradventure, dormant seeds of diathetic disorders, may have been put in process of germination, or the great organs of nutrition have become involved in lasting error of action. Let not the immediate danger obscure the remote, perhaps the greater. Very many diseases, by their occurrence and progress, give us the clue to the ultimate tendency to death. The "weak point" is disclosed; and the judicious physician, knowing this, may oftentimes so govern his patients' subsequent management of himself that he may, nevertheless, reach even unusual longevity.

As we have elsewhere, and often, insisted, a proper understanding of the action of the human body, and the causes of disease, will hereafter enable us to make use of the latter for the cure of other diseases, and even for securing increased longevity, both to the individual and the race.

After an introductory section (which we wish was more extended) with hints on mode of examination, general therapeutics, etc., the author treats *seriatim* of Simple Atrophy from Insufficient Nourishment, Chronic Diarrhœa, Chronic Vomiting, Rickets, Congenital Syphilis, Worms, Chronic Tuberculosis, Chronic Pulmonary Phthisis, Tuberculization of Glands, and an Addendum, discussing Derangements of the Bowels attendant on Second Dentition.

The diagnosis of each of these disorders is perspicuously made out, and but very little space devoted to pathological considerations. It is evidently a book that aims especially at *practicality*, and hence more attention is paid to formularized treatment than has been usual in these later years.

Numberless formulæ stand out in relief upon its pages. These are characterized by correctness of combination and pleasantness of form, rather than by any novelty. So far as we can judge, they are all of that character that may find occasional use. To younger practitioners, who seek a practical guide whilst learning how to treat the diseases of children, under the lead of wide and more comprehensive views, it will afford an assistance for which they may be temporarily grateful. We trust it may not fasten them to routine treatment, and superficial ideas.

*Manual of Hypodermic Medication.* By ROBERTS BARTHOLOW, A.M., M.D., Professor of Materia Medica and Therapeutics in the Medical College of Ohio, etc., etc. Philadelphia: J. B. Lippincott & Co. 1869. Pp. 150.

THIS is a very convenient little book, embodying the practical experience of its author in hypodermic medication, and referring, in suitable terms, to the contributions of French, German and English authorities, upon the same topic.

The first part treats of the History, Technology and General Therapeutics of the method. The second part gives illustrations of the use of Morphia, Atropia, Strychnia, Conia, Woorara, Nicotia, Hydrocyanic Acid, Physostigma, Caffein, Ergotin, Quinia, Mercury, Arsenic, and Irritant injections.

Plates of the instruments preferred are given, and also those diagrams illustrating the effect upon the pulse, temperature and respiration of subcutaneous injection of Morphia and Atropia, separately and combined.

For those who wish to make constant or occasional use of this method of securing the usual effects of active and yet concentrated medicines, we cordially recommend this little treatise as the best on the subject.

---

### Pamphlets.

*The Pathology of Bright's Disease.* By Wm. B. Lewis, M.D., Lecturer on Renal Pathology in the Medical Department of the University of the City of New York; Microscopist of Charity Hospital, etc.; with Illustrations. Published by Turner & Mignard, 109 Nassau Street, New York. Pp. 29. Price, 50 cents.

A valuable contribution, in brief, to the literature of the subject. The writer adopts Virchow's classification of kidney diseases: "An organic lesion affecting primarily the tubes and their lining; another producing at first increase and afterwards contraction of the fibrous stroma; and a third attacking the vascular supply of the parts," but observes: "If we accept a threefold division as the first law of renal pathology, the second is that one of these types of disease may excite another, so that it is not unusual to find two, or, apparently, even all of them coexisting in the same organ." Albuminuria is a broader term than Bright's disease. Renal congestion we are justified in classing (Dr. Wm. Roberts) as a separate morbid state included in the term albuminuria, but not in Bright's disease. The author disagrees with Dr. G. Stewart in his idea of "acute nephritis terminating fatally in the first stage," and gives several detailed cases, with illustrations from microscopic observations. These show the condition to be renal congestion, active or passive, and present no indications of inflammatory action, notwithstanding the presence of albumen, casts and even blood in the urine.

The discrepancy of view, after all, is merely one of definition — Dr. Stewart would include the *active* renal congestion as evidence of existent inflammation in its primary stage.

"The three types of morbid processes properly included in the term Bright's disease are as follows:

"I. Tubal nephritis, also called *acute desquamative nephritis, acute diffuse nephritis, the inflammatory form of Bright's disease, croupous nephritis, and acute Bright's disease.*

"II. Granular degeneration, also called *chronic desquamative nephritis, parenchymatous nephritis, the cirrhotic or contracting form of Bright's disease, the gouty, or the fibroid kidney.*

"III. The waxy, *depurative, amyloid, or lardaceous disease.*

"Authors who make the duration of the malady a basis for classification, include the second and third divisions under the term *chronic Bright's disease.*"

The main features of the three morbid processes classed under the head of Bright's disease are then examined in detail, and the results illustrated by diagrams from microscopic study, and the author concludes:

"We have now examined the main features of the three morbid processes classed under the title Bright's disease. It has been shown that tubal disease is an accompaniment of two forms, and the essence of the other; that in tubal nephritis, disease is confined to the tubes and their epithelium; in granular disease, primarily to the connective tissue, but that subsequently, tubal disease may appear; in waxy degeneration, primarily to the arterioles and capillary tufts, but that as a consequence tubal disturbance often manifests itself. Contraction and a surface covered, as it were, with granulations, are found to be characteristic of advanced granular disease, but appear also in waxy degeneration from similar causes. Fatty degeneration is reduced from the rank of a disease to that of a symptom; consequent upon sustained epithelial disturbance, it appears whenever this condition obtains.

"The 'large white kidney' is a result of tubal nephritis, which in its chronic stage has resolved itself into fatty degeneration. The 'small white kidney' may be a result of either granular or waxy change. Such are the combinations, and the successions generally observed. Waxy infiltration is said, however, to follow occasionally in the train of tubal nephritis.

"In conclusion we may allude to the sometimes difficult matter of determining the original lesion as we examine combined forms of kidney disease. We have seen that Stewart and others constitute a third or contracting stage of tubal nephritis. But such a state of the organs must be brought about by two processes, contraction of the fibrous element, and disease of the tubes. Which of these has caused the other cannot be determined by the microscope alone. It is a question for clinical observation to decide.

"Of itself tubal nephritis is not known to cause, or, in any great proportion of instances, to be followed by hypertrophy of the left ventricle of the heart, or valvular disease of the same organ, or cirrhosis of the liver. On the other hand these are the commonest attendants of granular disease. Hence, if we meet the combined form above mentioned with such concomitants, it is quite certain that the granular disease is the older lesion. So in waxy degeneration, if a contracted kidney is associated with a considerable infiltration of other organs, known to be liable to such change, it is not difficult to decide that any tubal disturbance discovered must be a result and not a cause of the renal lesion. Again, if it should be questioned whether the contraction of such a kidney were not due to granular degeneration, clinical observation would show that a large liver, a heart almost always free from chronic disease, and the presence of some exhaustive drain upon the blood, conditions not found with the granular kidney, point to a process of disease essentially different.

"In Bright's disease, then, there are included three types of morbid change, but in accordance with certain well-defined laws, these may be variously combined in the same organ."

*Nocturnal Enuresis and Incontinence of Urine.* By FREDERICK G. SNELLING, M.D. Turner & Mignard, 109 Nassau Street, New York. Pp. 18.

DR. SNELLING calls attention to the "random and slipshod manner" in which these difficulties are usually treated. After indicating a large number of direct and indirect causes, he says:

"It is apparent that diverse as these may be, they can only act by giving rise to one of three prime conditions, viz.:

"Atony or paralysis of the bladder itself, permitting over-distension, and resulting in stillicidium.

"Abnormal irritability and contractility of the bladder sufficient to overcome the resistance of the sphincter.

"Or, atony or paralysis of the sphincter vesica itself.

"These conditions may vary in degree; or in a given case two of the conditions may coexist, as, for example, paralysis of both bladder and sphincter."

After pointing out the sources of nervous supply of the bladder and sphincter, he calls attention to the fact that although sometimes the fear of corporeal punishment or ridicule may often suffice to supply the necessary nervous stimulus to control the difficulty, yet there may be an error of application, for there may be insufficiency of power or actual paralysis "*either of the cerebro-spinal fibres*, *of the sympathetic systems*, *or of the muscular* coats of the bladder itself."

Another difficulty is "that the nervous system may become *habituated to inattention*, as also, on the other hand, to *the*

*unconscious exercise of voluntary power.*" These also may be connected with *deficient co-ordinating* power. The voluntary effort to contract the sphincter may result in contraction of the muscular coat of the bladder.

The therapeutical suggestions are quite full. We quote or abstract.

In the very young, after removing all *possible* causes of reflex irritation, he has had much success with:

℞ Tr. Lyttæ, - - - - - - ʒ v;

Tr. Nucis Vomicæ, - - - - - - ʒ iij;

Vini Ferri Dulcis, - - - - - ʒ v;

M. — A half teaspoonful twice or thrice a day to a child five or six years old.

If there be much nervous irritability there may be combined with this a drachm or a drachm and a half of Magendie's solution of morphine; also

℞ Tr. Nucis Vomicæ, - - - - -
Tr. Ferri Acet., - - - - - aa ℥ iij;

M. — 10 or 15 drops to be given after breakfast, and twice during the day.

In these cases the urine does not retain its normal character. It is always more copious than natural, of lower specific gravity, lighter in color, and rarely contains the proper amount of characteristic organic ingredients. In a case presenting these characteristics, arising from inherent feebleness of constitution and highly sensitive nervous system, I have obtained complete relief from the above prescriptions, combined with a tonic —

℞ Syrup of the Hypophosphites, - - - - ℥ v;
Elixir of Yellow Peruv. Bark, - - - - ℥ v;

M. — A teaspoonful twice a day before meals.

These cases generally bear iron well.

The difficulty is more common in girls than boys. They should be urged to exercise the will during the day so as to accustom the bladder to the presence of the urine. Opium, hyoscyamus and conium are the best sedatives in this form of irritable bladder. The combination of cantharides with opium will greatly strengthen the resisting power of the sphincter.

In over-distension and stillicidium from more or less paralysis:

The catheter should be in constant use, and tannin, catechu, ergot, uva ursi, buchu, cubebs, nitrate of potash and opium should be used, combined with the cautious use of the cold douche or shower-bath to the sacrum; or a blister over the same part. The tannic acid acts as a strong astringent (I am not yet prepared to advocate its injection), the uva ursi as an astringent and exciter of the detrusores urinæ — the ergot exercises a most powerful influence over the unstriped muscular fibre; the buchu is a most efficient stimulant to the bladder, as well as a diuretic; the cubebs is a stimulating tonic with an especial tendency to the urinary mucous membranes; the nitrate of potash acts upon the internal coats of the bladder by rendering the urine more acid and stimulating; and opium has the most marked and singular effect of contracting the bladder and diminishing its calibre, of decreasing the secretion of urine, and of producing a tonic contraction of the sphincter vesicæ. These remedies may be used singly and in various combinations. An excellent one is —

| | | |
|---|---|---|
| ℞ | Elixir Aromat. Secalis, - - - - - | ℥ ss; |
| | Tr. Uva Ursi, - - - - - - | ℥ ss; |
| | Tr. Buchu, - - - - - - - | ℥ vi; |
| | Ess. Pyrolæ, - - - - - - | ʒ ij; |
| | Syrup Zingiberis, - - - - - - | ℥ j; |

M. — Half or one teaspoonful three times a day for an adult.

Were the action of the haschisch or cannabis indica more certain, I would recommend it in combination — but it may be used alone in doses of from fifteen to forty minims, carefully watched. Another efficacious combination is —

| | | |
|---|---|---|
| ℞ | Nitrate of Potash, - - - - - | gr. x; |
| | Tannin, - - - - - - - | gr. ij; |

M. — Pro dose. To be taken three times a day in simple syrup or solution.

Strychnine has been injected into the bladder with success in the proportion of 1 part to 1,000 parts of water.

Cases of renal calculus, or other diseases of the kidney, diseases of the uterus, vagina, rectum, prostate, etc., etc., require each their special treatment, of course, but about all of them will be more or less relieved by the exhibition of bland mucilaginous drinks, rest, the application of strong tinct. aconite over the pubes, and the careful avoidance of all the causes of increased acidity or other irritating properties of the urine.

Small doses of belladonna have a well-known power of allaying spasm and irritability.

In those cases, lastly, dependent on atony or paralysis of the sphincter vesicæ, the sheet anchor of treatment will be strychnine or nux vomica, variously combined with the tincture of cantharides, opium, morphine, or cannabis indica, iron and the vegetable tonics, as, for instance —

| | | |
|---|---|---|
| ℞ | Ext. Nucis Vomicæ, - - - - - | gr. viij; |
| | Oxydi Ferri Nigri, - - - - - - | ʒ j; |
| | Pulv. Quassiæ, - - - - - - - | ʒ j; |
| | Syr. Absinthe, - - - - - - - | q.s; |

M.—Ft. massa et in pil xlviij divide. One pill to be taken three times a day.

With this may be given also any one of the many so-called ferrated elixirs of bark. They are all of them mild and excellent tonics for children. If there be much nervous disturbance with it—

℞ Tr. Cantharidis, - - - - - - ʒ ss;

Tr. Hyosciami, - - - - - - ℥ ij;

M.—A half teaspoonful twice or thrice a day.

A blister to the sacrum will also sometimes have the most marked effect.

If there be also a condition of morbid irritability of the neck—

℞ Ext. Belladonna, - - - - - gr. iij;
Ext. Nucis Vomicæ, - - - - gr. ivss;
Phos. Acid. Dil., - - - - - ℥ j;

M.—Fifteen drops three times a day.

If from a difficult labor, causing paralysis of the bladder or sphincter vesicæ, from pressure—

℞ Ergot of Rye (Elixir Aromat.) - - - - ʒ iij;

Tr. Nucis Vom., - - - - - ʒ j;

Phos. Acid Dil. - - - - - - ℥ j;

M.—Fifteen drops three times a day.

Or—

℞ Strychniæ, - - - - - - gr. j;

Tr. Ferri sesqui-chlor., - - - - - ʒ ij;

Balsam Copaib., - - - - - ʒ j;

Infus. quassiæ, - - - - - - ℥ xij;

M.—Dose, an ounce three times a day.

Tannin may also be given in one-grain doses, in substance night and morning.

An excellent combination is—

℞ Fl. Ext. Cubebæ, - - - - - ℥ ij;
Fl. Ext. Buchu, - - - - - - ℥ iv;
Ess. Gaultheriæ, - - - - - q. s.;

M.—Fifteen to thirty drops every two or four hours.

The balsams have also been recommended by Mr. Chabrely, as, for instance—

℞ Styrax Balsam, - - - - - - ʒ iss;

Peruv. Balsam, - - - - - - ʒ iss;

Honey, - - - - - - - ʒ ij;

Pulv. Gum Acac, - - - - - - q. s.

M.—To make an electuary—a teaspoonful night and morning.

Or—

℞ Styrax Balsam, - - - - - - ʒ iss;

Balsam Tolu, - - - - - - ʒ ij;

M.—Ft. massa et div. in pil aa gr. v. to be silvered. Four to eight pills a day.

Benzoic acid has also been used with benefit in doses of three grains in pill, two or three times a day. Combined with the tonics, wine should be given, in small quantities, daily.

If these methods fail, electricity may be tried. A *jugum* never, but recourse may be had to a plan (first recommended, I think, by Sir Benj. Brodie, and quoted by Gross,) of tying under the penis, along the urethra, a piece of flexible catheter by adhesive straps applied from the glans toward the pubes, and to be removed and replaced when the patient urinates.

Zinc ointment and glycerine are the best local applications to allay and soothe the excoriation produced by the dribbling urine.

We regret that the author in his valuable and instructive paper has not referred more at large to the great number of cases which are readily relieved by the mineral acids. The old-fashioned muriated tincture of iron has cured more cases, " taken as they come," than any other one remedy. The liquor ferri per nitrat. is also very efficient. The latter, combined with tinct. cantharid., we have seen cure very many cases.

The reaction, specific gravity, etc., of the urine, should always be tested in these cases. It is rarely abnormally acid in children with this trouble, but if it is so, either in them or adults, the bromide of potassium will add to its laurels in use.

In cases involving want of contractility of the sphincter, the following formula, published in the *American Journal of Medical Science*, many years since, will often promptly control:

℞ Veratriæ, - - - - - -
Morph. Sulph., - - - - - - aa gr. x;
Cerat. Simp., - - - - - ℥j;
M. — Ft. ungt.
S. — Apply cautiously over the perineal region, or above the pubis, once or twice a day.

Local cauterization of the urethra will cure some cases.

Suppositors, deftly prepared, will cure others.

It is an exceedingly " nasty," and oftentimes obstinate trouble, and we thank Dr. Snelling for recalling professional attention to its causes and treatment.

*The Physiological Action and Therapeutic Uses of the Acidum Phosphoricum Dilutum.* By JUDSON B. ANDREWS, M.D., Assistant Physician in the New York State Lunatic Asylum. Read before the Oneida County Medical Society. 1869. Pp. 17.

AFTER discussing the supposed material relations of this acid to the nervous tissue ("chemical food"), Dr. Andrews gives the usual sphygmographic drawings, illustrative of its effects upon the pulse. He then details several cases wherein its efficiency as a nerve tonic and stimulant seemed quite apparent.

We append, as giving the gist of the matter, the concluding pages of the monograph:

"Cases are sometimes under treatment at the Asylum, and more frequently in private practice, especially from among literary, professional or business men, which are characterized by loss of mental power from excessive brain activity.

"The patient is languid, unable to perform mental labor with the usual facility, is nervous, at times fearful, timid and agitated; the memory is weakened, and permanent impairment seriously threatened. Examination reveals no organic lesion, but the symptoms are such as justly occasion alarm. Such cases have been improperly called by some recent writers cases of cerebral paresis, a term too strong in its import, but expressive of the great danger which impends. For the recovery of these cases, relaxation from business and labor, and the use of the phosphoric acid, combined with some suitable tonic, generally suffices.

"In cases where mental effort has been protracted till a sense of weariness renders its continuance difficult, a dose of the acid, from its stimulant effect, relieves fatigue and seems to invigorate the mental powers, and prepare the mind for renewed exertion.* In the night sweats attending consumption, and other exhausting diseases, this acid is employed with benefit, and has some advantages over the aromatic sulphuric acid, so generally used. It is much more agreeable to the taste, more likely to be tolerated, and does not constipate the bowels. The anti-scorbutic power of this acid is well settled. A marked case of purpura occurred in the Asylum recently. The patient had been an inmate for several months, and though eating the ordinary diet of the house, in which vegetables are

* A professor in one of our Medical Schools, in a letter to Dr. Gray, recently remarked: "Wonderful thing that phosphoric acid, and well named by me psychological lemonade. My lunch at noon (we dine at six) consists of rich cheese, bread, and a glass of phosphoric acid lemonade, and on that I have worked eight and nine hours a day, with my pen, for the past seven weeks in this hot weather, without headache or any depressi6n. I never take over fifteen drops, and only once a day, and when fatigued. It is wonderful how quick it climbs into the anterior lobes, scatters capillary congestion, and satisfies the hungry tissue with its own pabulum."

bountifully supplied, became scorbutic. The gums were red and spongy; there was lassitude, soreness of the muscles, and an eruption presenting the forms of petechiæ and vibices upon the anterior of the chest and the inner surface of the thighs.

"The patient was given the acid in half drachm doses, and in two weeks entirely recovered. In cases of anæmia and chlorosis, in both of which there is a depressed condition of the nervous system, phosphoric acid in combination with ferruginous tonics, has been found especially efficacious.

"At the Asylum, as an adjuvant for the solution of quinia in water, phosphoric acid is now substituted for the sulphuric acid, with the advantage of increasing the tonic properties of the solution. In giving quinia, we have in cases marked by great nervous prostration, and accompanied with profuse perspiration, found this acid in half drachm doses a valuable addition, and one that seems to increase the power of the alkaloid. To the ordinary elixirs, tinctures and decoctions of bark, the acid forms an important aid, and by its acidity it overcomes to a great degree the unpleasant taste of the vegetable bitters.

"Observation here confirms the views of Nelligan and others, that this substance exerts no direct influence on the generative function. It has thus been employed on theoretic grounds; but any favorable influence it has exerted has probably been owing to its general tonic effect. We have used it extensively, and in cases where this function was abnormally excited; and in no instance has its administration been suspended from this cause, or has any inconvenience resulted from its use.

"Phosphorus in substance is now recommended in many of the Journals, in some forms of paralysis, in locomotor ataxy, and in other of the neuroses. It is an element difficult to dispense and dangerous to administer. In the stomach it is converted largely into phosphoric acid. It is from this change taking place in the stomach, that the danger is to be apprehended. Is it not better to employ the acid, which in proper doses is harmless, than to incur the risk of consequences in giving phosphorus in substance?

"In the administration of this remedy, one general principle should be kept in mind, viz.: not to exhibit it in cases of congestion of the brain, or in those in which there is an inflammatory action, either in the nerve substance or the meninges, as its stimulant effect might prove an aggravation to existing disease. In no case in which it has been given, has it disturbed digestion or proved an irritant to the stomach, even when its administration has been prolonged.

"When the remedy was first employed at the Asylum, the dose was ten drops three times a day; this has been gradually increased till now the standard dose is a half drachm. This is given in water alone, or with simple syrup, with which it makes a pleasant and agreeable lemonade. The large doses spoken of were thus taken. In the various combinations with other remedies, the dose varies from 10 to 20 drops. A favorite tonic remedy, which makes an eligible preparation, and one very palatable, is as follows:

| | | |
|---|---|---|
| ℞ | Acidi Phosphorici Dil., - - - - | one oz. |
| | Elix. Calisay., - - - - - - | four oz. |
| | Elix. Valer Ammon., - - - - - | two oz. |
| | Glycerinæ, - - - - - - - | three oz. |
| | Sherry Wine, - - . - - - | six oz. |

Dose, from one-half to one oz.

It is from an experience in the use of this remedy in more than two hundred cases, extending over a period of several years, that confidence has been inspired in its general adaptation to the treatment of diseases marked by debility of the nervous system.

*Memoir of Robley Dunglison, M.D., LL.D.* Read before the College of Physicians at a special meeting held October 20, 1869. By S. D. Gross, M.D., LL.D. Philadelphia. 1869.

*Biographical Sketch of the late A. B. Shipman, M.D., of Syracuse, N. Y.* Read before the Onondaga Medical Society. By H. O. Jewett, M.D., Cortland, N. Y. Albany. 1869.

Earnest, eloquent and worthy tributes to medical men whose names the profession "will not willingly let die."

*Aiken; or, Climate Cure.* By Amory Coffin, M.D., and W. H. Geddings, M.D. Charleston, S. C. Pp. 58.

This pamphlet is sent forth with the avowed purpose of convincing the reader that the climate of Aiken is peculiarly advantageous, in a sanitary point of view, as a resort for Northern invalids during the winter months. Its reputation in this respect was steadily increasing up to the commencement of the war, when, of course, "circumstances prevented."

The authors do not confine themselves to bare assertions or cataloguing instances of cure or relief, but bring forward carefully prepared meteorological tables, which include a period of eight years. Without going into detail here, we quote the statement:

"The atmosphere is so dry that surgical and other instruments, guns, etc., which require so much care in other places to prevent their rusting, may be exposed here for months without sustaining damage."

Wells have to be dug from 90 to 150 feet. The soil is porous, and the heaviest rain dries off in a few hours. "Fogs are extremely rare, and the Epiphyte Tillandsia, or tree moss, that unfailing indicator of moisture and malaria, which so gracefully festoons the live oaks of the low country, is entirely absent."

The mean annual temperature is 61° 69. The mean temperature of the colder six months of the year is 51° 53.

This is about as favorable an exhibit as can be given by the most vaunted resorts in Europe—Nice, Palermo, Pau, Venice, Pisa, etc.

The winds are mainly from the southwest, and very moderate. Southeast winds are rare. From the combination of dryness, mild temperature and freedom from injurious winds, the patient, it is claimed, can pass the greater portion of his time in the open air, and *scarcely know what confinement indoors means.* In the 55 days prior to writing there were only three when he could not have been out of doors enjoying the bright, warm sunshine and balmy air.

The *elevation* is only 600 feet above the sea level—the advantage of this in cases where there is a tendency to hæmorrhage is obvious. Practically, it is asserted, the climate proves itself tonic and bracing, rapidly promoting convalescence from tedious diseases, and peculiarly beneficial in phthisical cases. Its tonic influences can not be experienced in the mild but debilitating climate of Florida or Cuba.

Aiken is a town of about 1,300 inhabitants, 120 miles northwesterly from Charleston, accessible by rail. It is but 17 miles from Augusta, with which the same railroad connects it.

We have devoted some space to this pamphlet, because we of this section need some accessible point where we can send patients into a climate possessing exactly the peculiarities claimed for Aiken, South Carolina.

*Comparison of the Mortality from Disease in Armies with that of Men of Military Ages in Civil Life, showing the Groups of Diseases chiefly concerned in Causing the Excess of Mortality in Armies.* By A. NEWMAN, M.D., Lawrence, Kansas. Leavenworth. 1869. Pp. 39.

A large amount of valuable and instructive matter has herein been industriously collected by the author, but it has come to hand too late for analysis at present. We regret that competent medical men should seek the limited and temporary circulation afforded by the monograph, rather than the wide diffusion and permanent preservation afforded by the leading medical journals.

